# Book Review

**Published:** 1896-12

**Authors:** 


**The History of Surgical Anæsthesia.** By H. Bellamy Gardner. Pp. 31. London: Baillière, Tindall & Cox. [1896]. —This is a paper read before the Medical Society of Charing Cross Hospital, and is opportunely published at the present time. It is interesting historically, and shows much research on the author's part.

**Prostitution in Antiquity: in its Connection with Venereal Diseases.** By Dr. Edmond Dupouy. Translated by Thos. C. Minor, M.D. Pp. 93. Reprinted from *The Cincinnati Lancet-Clinic.* 1895.—This "Study in Social Hygiene, written for medical brothers, physicians habituated, like the author, to seeing the different pictures of human pathological anatomy, physically and morally," gives a graphic description of the worship of Venus and Phallus by the men and women of Corinth; of the sanguinary satyrs who were formerly known as emperors, senators, and nobles of Rome; of the worship of Priapus, Isis, and Osiris in Egypt; of the cult of Lingam in India; of the worship of Baal amongst the Hebrews; and of the wide distribution of urethral and venereal diseases throughout all times.

**Diets for Infants and Children in Health and in Disease.** By Louis Starr, M.D. Philadelphia: W. B. Saunders. 1896.— This is a very useful pocket-book with detachable leaves, on which are printed forms to be filled up with the appropriate diets for children of all ages.

**The Eye in its Relation to Health.** By CHALMER PRENTICE, M.D. Pp. 214. Bristol: John Wright & Co. 1895.—It is not easy to see for what purpose this book was written. "Eye-strain" is a factor of some physiological import; but its importance has been so ridiculously exaggerated by the author that the book is likely to do more harm than good in the cause it is intended to support. Every conceivable disease is set down as depending on eye-strain: diabetes, consumption, ovaritis, insanity, etc., etc. The author's opinion is that "so-called cures for dipsomania perform whatever good they effect by temporarily relieving eye-strain." The book might amuse or seem very clever to a lay reader; but to a scientific mind its pretensions are absurd. It is true that it lies on a substratum of truth, based upon the work of George Stevens on the anomalies of the ocular muscles.

**The Medical Inspection of, and Physical Education in, Schools.** By CHARLES ROBERTS. Pp. 24. London: John Bale & Sons. [1896.]—This is a reprint of the paper prepared by the author for the Royal Commission on Secondary Education, and issued in the Blue-book containing the fifth volume of their Report. The points dealt with are well worthy the careful attention of medical officers of schools, of schoolmasters and schoolmistresses, amongst whom in its present accessible form it should find a large circulation.

**King's College Hospital Reports.** Volume II. London: Adlard and Son. 1896.—This volume should well maintain the interest of old King's College students in the work still done in their former school; it contains original articles and summaries of what has been passing in the various departments of the Hospital during the past year. There are reports with statistical tables bearing upon every special branch of our work, each of them well worthy of perusal, as are the "Notes on Cases of Interest" reported by various members of the staff. The original papers are excellent.

**London Temperance Hospital: Report upon the Surgical and Ophthalmic Cases admitted during the Year 1894.** London: J. Vincent and Son. [1895.]—This compilation consists solely of a number of tables and statistics, and is a proof, where proof is scarcely needed, that good work is being done at the London Temperance Hospital, the physician to which was Sir B. W. Richardson, whose recent death is regretted by the profession at large. For those who have access to the hospital notes the publication affords a convenient index, but it is scarcely more than this. In the table of "special operations," which includes three ordinary operations for excision of varicocele, a short column of "remarks" in very condensed English does not afford sufficient information for safe deductions to be formed as to the general value of the various surgical procedures adopted. In the portion devoted to ophthalmic operations (which we notice includes all the period from 1888 to 1894) the information is still more deficient. In hardly any case is the appearance or maturity of the lens specified, and in the table of "Hard Cataracts" the "remark" *hard cataract* or *senile cataract* does not convey further information. In the table of "Soft Cataracts" (which, by the way, contains a Morgagnian cataract), the "result" in each case is left blank. Only twenty per cent. of the extractions required subsequent discission, but why the proportion is so small is left a matter of conjecture.

**Transactions of the Royal Academy of Medicine in Ireland.** Vol. XIII. Dublin: Fannin and Co., Ltd. 1895.—This volume might well be known as a small-pox number, inasmuch as there are no less than three papers on this subject. Dr. J. W. Moore advocates the complete exclusion of daylight, or the use of tightly-closing red curtains or windows of red glass for the purpose of excluding the actinic rays. These rays are also responsible for Dr. H. C. Tweedy's case of argyria, after five years' treatment of locomotor ataxia by nitrate of silver pills; the patient continued to take the silver salt at intervals for twenty-three years, and he must have taken more than two thousand grains of the drug. We may add that the mention of the chemical action of light rays has not in this case led up to the new photography, for there is no skiagram in the volume.

**Transactions of the American Gynecological Society.** Vol. XX. Philadelphia: Wm. J. Dornan. 1895.—This volume contains many valuable papers on such vexed questions as hysterectomy for fibroids, the value of Alexander's operation, deciduoma malignum, and other subjects of interest to the abdominal surgeon and gynæcologist. The discussions which followed the reading of these papers are given in full, and we may therefore regard the work as containing the latest views of most of those who have made American surgeons so justly famous in this department. Its pages are full of the practical thoughts of practical men, and there is scarcely an article that can be read without profit. The book is well bound, and contains some well-executed plates. In addition to its own index, the volume contains a most useful index to vols. i. to xx.

**Transactions of the American Pediatric Society.** Vol. VII. New York: Rooney & Otten Printing Co. 1895.—In this country we have no society to publish cases of children's diseases which are worthy of being recorded, and the American Pediatric Society, consisting of members from the States and the Dominion, is to be congratulated on the useful publication it has sent forth. All the papers are of great practical interest and well worth careful perusal. It opens with a notice of the poem of Hugh Downman, parson and doctor of Exeter in 1770, on "Infancy," in six books in iambic pentameters. The poem is curious and shows the very sound knowledge held on the bringing-up of children, though the information is veiled in somewhat pedantic verse.

**Transactions of the Ophthalmological Society of the United Kingdom.** Vol. XV. J. & A. Churchill. 1895.—This volume resembles its predecessor in containing a number of very valuable papers, some of them abtruse, others well worthy of the perusal of every practitioner. It commences with Gowers's valuable paper on "Subjective Visual Sensations," particularly bearing on epilepsy and migraine; there is a most exhaustive report of a case of filaria, too, by Argyll Robertson. The disease seems to be confined to the West Coast of Africa, particularly the region of Old Calabar. It would appear that the worm passes from the blood to the eye, and not from without through the conjunctiva or skin. The movements of the worm are pretty extensive, at any rate it passes with ease from one eyeball to the other. Lawford has papers on "Hydatid Cyst of Orbit," and on "Ophthalmia Nodosa," which latter usually follows injury to the eye by a caterpillar of the family Bombycidæ. Treacher Collins gives several papers; one on "Lamellar Cataract and Rickets," quoting the opinions of several oculists in Australia, where rickets and lamellar cataract are both rare. Other equally meritorious papers are contained in the book, which is well worthy of careful perusal, and is very well published.

**Transactions of the Dermatological Society of Great Britain and Ireland.** Vol. I. London: H. K. Lewis. 1895.—This volume is the result of a new departure. As the Dermatological Society of London was considered too exclusive, the Dermatological Society of Great Britain and Ireland was started in 1894. Dr. Pye-Smith's presidential address was able, comprehensive, and very interesting. At the first Congress of this Society, Dr. Byrom Bramwell gave an address on the thyroid treatment of skin diseases; at the other meetings many interesting papers were read and cases shown. We must not expect too much from the first issue of a new society, but for one with such a comprehensive name 91 pages are a little mean for the record of a year's work, and we hope to see future volumes enriched with appropriate illustrations.

**Transactions of the American Laryngological Association.** New York: D. Appleton and Company. 1896.—From cover to cover this volume is an unbroken record of valuable work, for the most part by American rhinologists whose names are familiar to rhinal specialists on this side of the Atlantic. One of the most useful and original groups of articles is that which refers to suppurative accessory sinus disease, and the contribution on tuberculous disease of the upper air passages is very complete.

**Transactions of the Epidemiological Society of London.** New Series. Vol. XIV. London: Shaw and Sons. 1895.—The contents of this volume maintain the high average which former issues have led us to expect. Dr. Shirley Murphy's presidential address covers a wide field in discussing in practical and suggestive manner "The Study of Epidemiology," and Dr. Klein contributes a paper of special interest on "The Relation of Bacteria and their Toxines." A clear and excellent photograph of the late Sir George Buchanan forms a fitting frontispiece to the volume, and the Obituary contains an appreciative notice of this distinguished Chief of the Medical Department of the Local Government Board.

**The Laryngoscope.** St. Louis.—We gladly welcome this monthly addition to our Exchange-list. It began its existence in July, and has for its editors Dr. Frank M. Rumbold and Dr. M. A. Goldstein, who are supported by an influential staff of assistant and foreign editors. We are told that it is the intention of the new journal to present to the profession the essence of practical experience of those interested in diseases of the nose, throat, and ear. It is not published solely for the benefit of the specialist, but for the "busy and ever-advancing general practitioner" as well. The first number leads off with a very clear and practical article on the photo-fluoroscope, by Dr. J. Mount Bleyer, with excellent illustrations of the apparatus, and of skiagrams of intubation tubes in the larynx and the trachea; hay fever is dealt with by Dr. Seth Scott Bishop; lingual tonsils by Dr. E. R. Lewis; then follows the first part of the clinical investigation of ear diseases, by Dr. Dundas Grant; Dr. Lederman discusses petro-mastoid necrosis; Dr. J. E. Schadle, post-nasal adenoids. The rest of this excellent number is taken up with descriptions of new instruments and extracts from current literature. The second number quite maintains the high standard of the first. The editors are much to be congratulated on their first endeavours, and if future numbers are equal to the early ones, *The Laryngoscope* deserves a successful and prosperous career.

**Letts's Diaries—Office, Pocket, and Medical Editions for 1897.** London: Cassell & Company Limited.—Letts's Diaries have stood the test of many years' experience, and are still in high favour. We are very pleased with the assortment we have just received. No. 8 Office Form, containing one day only on a page, should afford ample opportunity for all diarists, except the most prosy. A special feature of the book is a tablet on the inside of the back cover for receiving notes with slate or lead pencil, which can be easily erased. Two Pocket Editions, Nos. 20 and 22, are specially attractive. They have a week in an opening and are fitted into most tempting wallets, with receptacles in one case for papers and in the other for cards, stamps, &c. The Medical Diaries are handsome productions and are in two sizes, one with space for 54 patients, and the other for 108. They offer many conveniences. The tendency of these things is to get too bulky, and we should prefer to see these particular issues without the advertisements which are bound in with them. We have received from the same firm a sample of their Medical Prescription Copyist, one of the well-known forms for using with carbon paper. It is the least attractive in outward show, but is no doubt intended to be received into a case, to which the price on the cover probably refers, although this is left to be guessed.

**Visiting List.** Bristol: John Wright & Company. 1897.—This list has again been improved in minor details, and is now issued in three forms: *A*. Monthly, with fixed dates. *B*. Perpetual. *C*. A new weekly list arranged for 80 patients. *A* and *B* arranged as monthly lists have proved to be most convenient, complete, and time-saving: they avoid the necessity for re-writing names of chronic cases every week. The device of ruling each alternate line in red ink is very convenient for rapid reference. A pencil has now been added, and the lists appear to be as complete as ingenuity can make them.

**Sixty-first Annual Report of the British Medical Benevolent Fund, for the Year 1895.** London: Morton & Burt. — It may be convenient here to re-state that the objects of this fund are: (1) To afford immediate pecuniary relief to distressed qualified members of the medical profession, their widows or orphans. (2) To grant annuities to such after they have attained sixty years of age. In 1895 there were 212 applications for grants, but only 158 of these cases could be relieved. Amongst them the sum of £1,548 was distributed. The annuitants number 107, and receive altogether £2,155. We are grieved to see Bristol so poorly represented in the subscription list. We miss several well-known names. Surely it should be the exception and not the rule for a medical man not to subscribe to this excellent fund, so carefully and wisely administered.

A new journal is announced for 1897, "in which Scottish work and teaching in all departments of Medicine are to be more fully represented than they have hitherto been." With a large staff of contributors from Edinburgh, Glasgow, Aberdeen, and Dundee, this new monthly should have a brilliant future. It is to be called *The Scottish Medical and Surgical Journal*, and is to be published by Mr. William F. Clay, of Edinburgh.

The *Journal of Nervous and Mental Disease* announces that its Editors are now Dr. Chas. L. Dana, Dr. F. X. Dercum, Dr. Philip Coombs Knapp, Dr. Chas. K. Mills, Dr. Jas. J. Putnam, Dr. B. Sachs, and Dr. M. Allen Starr. The address of the Managing Editor, Dr. Chas. Henry Brown, is 25 West 45th Street, New York.

